# Long-term administration of bumetanide improve functional recovery after spinal cord injury in rats

**DOI:** 10.3389/fphar.2022.932487

**Published:** 2022-10-19

**Authors:** Shiva Hashemizadeh, Zeinab Gharaylou, Saereh Hosseindoost, Maryam Sardari, Ameneh Omidi, Hassan Hosseini ravandi, Mahmoudreza Hadjighassem

**Affiliations:** ^1^ Brain and Spinal Cord Injury Research Center, Neuroscience Institute, Tehran University of Medical Sciences, Tehran, Iran; ^2^ School of Advanced Technologies in Medicine, Tehran University of Medical Sciences, Tehran, Iran; ^3^ Shefa Neuroscience Research Center, Tehran, Iran; ^4^ Pain Research Center, Neuroscience Institute, Tehran University of Medical Sciences, Tehran, Iran; ^5^ Department of Animal Biology, School of Biology, College of Science, University of Tehran, Tehran, Iran; ^6^ Department of Anatomical Sciences, Faculty of Medical Science, Tarbiat Modares University, Tehran, Iran

**Keywords:** bumetanide, contusion, NKCC1, rat, spinal cord injury

## Abstract

Ion disturbances are among the most remarkable deficits in spinal cord injury (SCI). GABA is an integral part of neural interaction. Action of the GABA_A_ receptor depends on the amount of intracellular chloride. Homeostasis of chloride is controlled by two co-transporters, NKCC1 and KCC2. Previous studies revealed that NKCC1 are disturbed in SCI. In this study, NKCC1 is highly expressed in the epicenter of the lesioned spinal cord at 3 hours after induction of the lesion and reached the peak around 6 hours after SCI. Bumetanide (2 and 4 mg/day), as a specific NKCC1 inhibitor, was used at 3 hours post SCI for 28 days. The functional recovery outcomes were measured by the Basso–Beattie–Bresnahan (BBB) locomotor rating scale, ladder walking test, and hot plate test. The rats that received bumetanide 4 mg/day exhibited improved recovery of locomotor function, reduction of NKCC1 gene expression, and upregulation of GAP protein levels 28 days post SCI. Histological tissue evaluations confirmed bumetanide’s neuroprotective and regenerative effects. This study provides novel evidence for the benefits of bumetanide in early administration after SCI.

## 1 Introduction

Spinal cord injury (SCI) causes damage to the axonal pathways controlling sensory and motor function (O'Shea et al., 2017). Delayed secondary damage following primary injury to the spinal cord is responsible for progressive degeneration of the structure and results in neurological impairment below the injury level. The secondary injury phase consists of vascular change, ionic-imbalance, excitotoxicity, oxidative stress, and inflammation that lead to further neuronal and glial loss and scar formation (Quadri et al., 2020).

Early management of acute SCI is much more complex (Sánchez et al., 2020). Current standard strategies, including early decompression surgery, acute immunosuppressive with high-dose methylprednisolone (MP), and later rehabilitation therapy, alleviate spinal damage and improve patients’ quality of life (Galeiras Vázquez et al., 2017; Hachem et al., 2017). In contrast, MP administration may increase the risk of adverse events in SCI patients, such as infection (Liu Z. et al., 2019b). The pharmacological approach applied to treat SCI targets secondary cascades to reduce neuronal cell loss and preserve motor function (Karsy and Hawryluk, 2019). Thus, recent advanced research focused on discovery of novel therapeutic agents to overcome the complexity of the secondary mechanism (Sun et al., 2018; Venkatesh et al., 2019; Zavvarian et al., 2020).

Ion disturbances are one of the first events that occur after SCI. Dysregulation of ionic homeostasis plays an essential role in pathological changes post SCI, resulting in edema and excitotoxicity (Kwo et al., 1989). Specifically, intracellular or extracellular accumulation of Na+, K+, Ca^2+^, and Mg^2+^ ions disrupts ion gradients, which is associated with impairment of specific ion channels and related transporter activity (Young and Koreh, 1986; Young, 1992). So, based on these findings, ion channel blockers are possible strategies to treat SCI-induced dysfunction (Liu et al., 2011; O’hare Doig et al., 2020).

The inwardly directed Na ^+^-K ^+^-Cl^-^ co-transporter isoform 1(NKCC1) is present in neuronal and non-neuronal cells (Luo et al., 2021) and plays a vital role in maintenance of ion hemostasis (Zhang et al., 2021). Previous studies have demonstrated the role of NKCC1 in neurological diseases, including epilepsy (Liu R. et al., 2019a; Gharaylou et al., 2019b), neuropathic pain (Zarepour et al., 2020), traumatic brain injury (Zhang et al., 2017), stroke (Huang et al., 2019), neurodegenerative and psychiatric diseases (Hadjikhani et al., 2018), and SCI (Huang et al., 2021). Importantly, bumetanide is a selective NKCC1 inhibitor approved by the U.S. Food and Drug Administration (FDA) to treat cardiac failure and renal disease (Sidhu and Puckett, 2021). Recent work demonstrated that acute high-dose pharmacological blockade of (30 mg/kg) of NKCC1 is essential to H-reflex recovery after the transection SCI model (Côté et al., 2014). Moreover, pretreatment with low-dosage (0.3 mg/kg) NKCC1-blockade combined with the blocking of AQP4 reduces edema and tissue loss following spinal cord injury (Yan et al., 2018). Clinically speaking, the optimal time for treatment to target secondary cascades is a crucial consideration following SCI. However, no experimental data are currently available to examine the timing of the beneficial effect of bumetanide treatment to inhibit NKCC1 transporters after SCI. Therefore, first, we select the optimal treatment time depending on the expression level of NKCC1. In this study, we determined the time course of NKCC1 expression following induction of SCI and provide the best starting time to use bumetanide.

## 2 Materials and methods

### 2.1 Animal preparation

**FIGURE 1:**
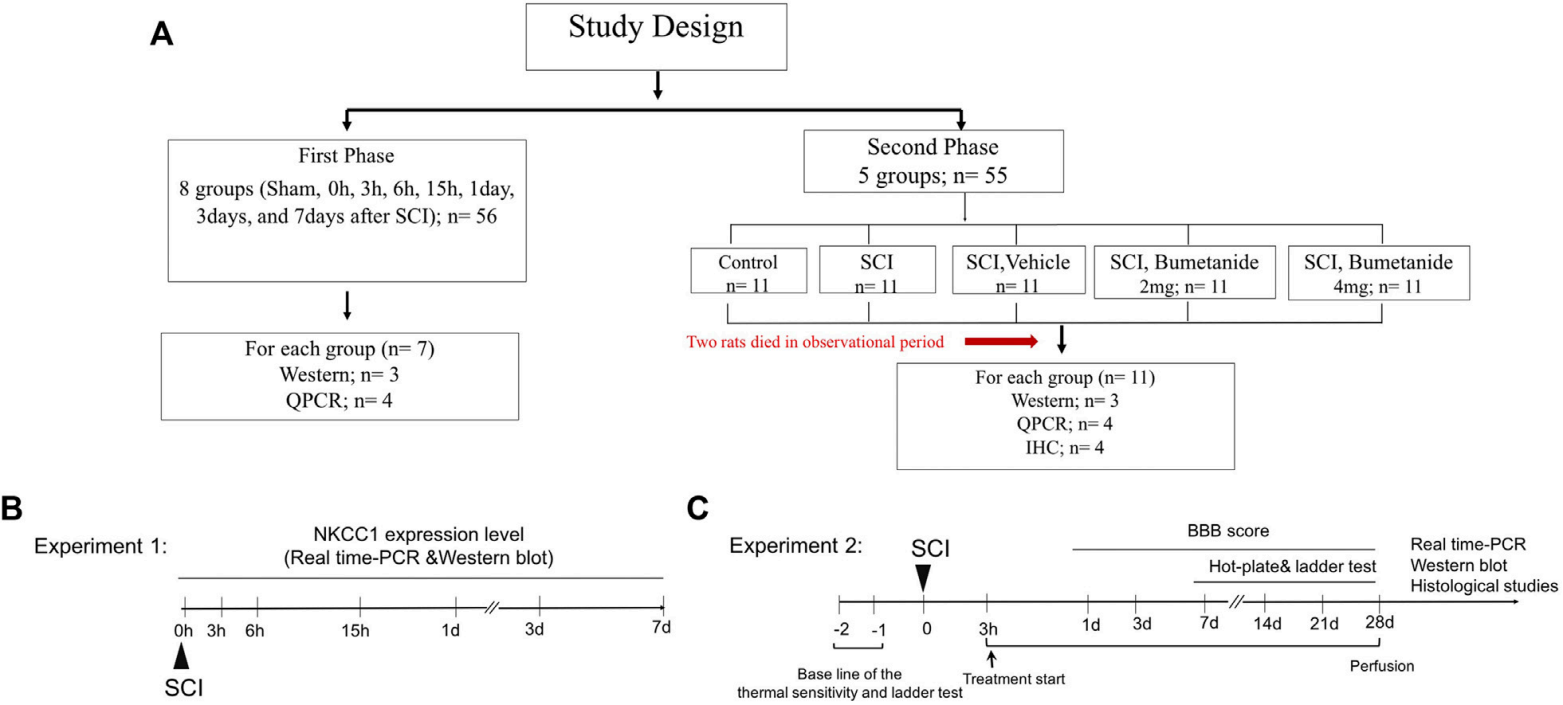
**(A)** Experimental plan and treatment groups. **(B)** Experiment 1 designed to indicate time course for NKCC1 expression change. Rats were subjected to the spinal cord injury and rats were sacrificed in 0, 6, and 15 h, 1, 3, and 7 days after SCI and subjected to the NKCC1 expression level analysis. **(C)** experiment 2 designed to determine the therapeutic effect of bumetanide .Rats were treated by bumetanide or vehicle for 28 days, and rats of each subgroup were successively performed with behavioral test, and the subsequent decapitation for molecular and immunohistochemical analysis 4 weeks after SCI.

Male Wistar adult rats weighing 250 ± 10 g were used for all experiments. The rats were group-housed in a temperature-controlled room (22 ± 2°C) and maintained on a 12-h light-dark cycle with *ad libitum* access to water and food. All animals used in the study were obtained from the Faculty of Pharmacy, Tehran University of Medical Sciences. All procedures of animal care and protocols were confirmed by the Animal Ethics Committee of Tehran University of Medical Science Center (Protocol number: IR.TUMS.VCR.REC.1397.904) and conducted in accordance with the Guiding Principles for the Care and Use of laboratory animal and followed the ARRIVE 2.0 guidelines (Percie Du Sert et al., 2020) (Figure 1A).

### 2.2 Experimental design

#### 2.2.1 Experiment 1: time course for NKCC1 expression change

We want to evaluate the beneficial effect of bumetanide as a selective NKCC1 inhibitor after SCI. Twenty-four rats were randomly selected to establish a contusion spinal cord injury model. The injured segment at 0 h, 3 h, 6 h, 15 h, 1 day, 3 days, and 7 days after SCI was evaluated to determine the changes in NKCC1 mRNA and protein levels within the lesion center using real-time polymerase chain reaction and Western blot, respectively. The appropriate time for bumetanide treatment was selected based on the NKCC1 expression change (Figure 1B).

#### 2.2.2 Experiment 2: determination of the therapeutic dose

Adult male Wistar rats were randomly divided into five groups as follows: Group I: intact (control); Group II: rats underwent SCI induction without receiving any treatment (SCI); Group III: SCI treated with intraperitoneal injection of bumetanide 2 mg/kg started at 3 h after SCI and subsequently every 24 h for 28 days (SCI+Bum 2 mg/kg); Group IV: SCI treated with intraperitoneal injection of bumetanide 4 mg/kg started at 3 h after SCI and subsequently every 24 h for 28 days (SCI+Bum 4 mg/kg); and Group V: SCI treated with ethanol 3 h after SCI and subsequently every 24 h as a vehicle (SCI+ vehicle) (Figure 1C).

### 2.3 Bumetanide injection

Bumetanide (Sigma-Aldrich, B3023) was dissolved in 50 ml ethanol and then diluted in 0.9% saline. Bumetanide was administrated via intraperitoneal injection starting at 3 h after SCI and then daily until 28 days, while the vehicle-treated rats received the same volume of ethanol in saline (Figure 1).

### 2.4 Spinal cord injury model

Animals were subjected to SCI using a contusion injury model. All surgical procedures were conducted under aseptic conditions. The rats were anesthetized with an intraperitoneal (i.p) injection of ketamine and xylene and checked for pain reflexes. Under deep anesthesia, their spinal cord was exposed by dorsal laminectomy at the T9–T11 level. For the contusion injury, we applied a custom-made device model (Borj Sanat company, Iran). A 10-g metal rod was dropped from 25 mm height through a guide tube positioned perpendicular to the exposed spinal cord (Hashemizadeh et al., 2021). After the contusion injury, the muscles and skin were sutured using 3–0 silk sutures. The animals were placed in a temperature-controlled room. Manual expression of the bladder was applied twice per day until spontaneous reflex was observed. Antibiotic therapy was given for 5 days to avoid complications. Under these conditions, two SCI rats were found dead after 10 days, one (vehicle-treated) rat after 7 days, and one (bumetanide 2m/kg- treated) after 14 days post SCI. One rat in the bumetanide 2m/kg-treated group died early in the experiment due to bladder rupture during the early post-operative care regimen. Under these conditions, no animal had to be killed due to sickness or urinary infection.

### 2.5 Real-time reverse transcriptase-polymerase chain reaction

Total RNA was isolated from the center of the lesion or the uninjured spinal cord using TRIzol (Sigma). The first-strand cDNA was synthesized with the TaKaRa Prime Script RT reagent Kit (TaKaRa Bio, Kusatsu, Japan). According to each manufacturer’s protocol, real-time PCR was performed using BioFACT™ 2X Real-Time PCR Master Mix (High ROX) with an ABI system. Real-time PCR was conducted in triplicate with each RNA sample at 95°C for 15 min, followed by 45 cycles of 15 s at 94°C, 60°C for 15 s, and 72°C for 30 s in sequence. The forward and reverse primers used for RT-qPCR were designed using the online software NCBI/Primer-BLAST. GAPDH was used as an internal control. The relative expression level of the target gene was analyzed using the ΔCt method. Primer sequences used for RT-qPCR:

NKCC1-F: 5′-GAT​ACT​AGG​GCA​TGG​GCT​GA-3′
NKCC1-R 5′-GGT​GCG​TTG​AGA​TCC​ATT​TT-3′
GAP-43-F:5′-CGACAGGATGAGGGTAAAGAAGA-3′
GAP-43-R: 5′-GTG​AGC​AGG​ACA​GGA​GAG​GAA-3′
GAPDH-F: 5′-CGT​GTT​CCT​ACC​CCC​AAT​GT-3′
GAPDH-R: 5′-TGT​CAT​CAT​ACT​TGG​CAG​GTT​TCT-3′

### 2.6 Immunoblotting assay

Spinal cord segments at the middle of the lesion site (1 cm approximately) were collected and homogenized with a cold RIPA lysis buffer containing 1 mM proteinase inhibitor. The lysate was centrifuged at 13,000 x g at 4°C for 20 min, and the supernatant was collected. The protein concentration was measured according to Bradford’s method. Equal amounts of the protein (40 μg per well) were separated on a 10% SDS-PAGE and transferred onto polyvinylidene difluoride membranes. Then, the membranes were blocked with 7% bovine serum albumin (BSA) in TBS-T (Tris-buffered saline containing 0.1% Tween 20) for 2 h at room temperature. After blocking, the membranes were incubated with primary antibodies against NKCC1 (Gharaylou et al., 2019b) (Abcam, ab59791, 1:2000 in 3% BSA/TBS-T) and GAP-43 (Asgari Taei et al., 2021) (Cell Signaling Technology, 8945, 1:1000 in 3% BSA/TBS-T) overnight at 4°C. After washing with TBS-T six times for 5 min each, the membranes were incubated with the HRP-conjugated secondary antibody (anti-Rabbit, Abcam, ab6721, 1:5000 in 3% milk/TBS-T) for 1 h at room temperature and then washed six times with TBS-T for 5 min each. Immunoreactivity was detected by an ECL kit (Amersham Biosciences, Freiburg, Germany) on radiography films (Fujifilm, Madrid, Spain). The membranes were stripped and incubated with a primary antibody against β-actin (Padza, PB 103, 1:2500 in 3% BSA/TBS-T). The densities of each band were standardized to their corresponding β-actin levels. Finally, a densitometry analysis was conducted by using ImageJ analysis software (Hosseindoost et al., 2020).

### 2.7 Behavioral assessments: Basso–Beattie–Bresnahan score, thermal sensitivity evaluation, and ladder test

#### 2.7.1 Basso–Beattie–Bresnahan score

Hind-limb motor function was assessed by using the Basso–Beattie–Bresnahan (BBB) locomotor rating scale developed by Basso et al., in which the score ranges from 0 (fully paralyzed) to 21 (complete functional recovery). The BBB score was recorded before surgery and at days 1 and 3 post injury and subsequently once per week for 28 days after SCI. The rats were assessed for 4 min in an open field, and hind-limb motor function was scored by two individuals independently, blinded to the experimental groups to avoid interference. The left and right hind limb scores were averaged to obtain a single value. In this experiment, scoring differences between two blinded independent investigators were low (0–1), and if there was disagreement in the BBB score, the lower score was recorded for analysis (Teixeira et al., 2018).

#### 2.7.2 Pain-related behavior: thermal sensitivity evaluation (hot plate)

The effect of bumetanide treatment as a selective inhibitor of NKCC1 on thermal sensitivity was evaluated by the hot plate test once a week for 28 days (Borj Sanat, Tehran). The hot plate test was used to assess thermal nociception. Briefly, all groups of rats were habituated to room temperature test 1 h before the test. During the tests, the rats were placed on a metal surface having a constant temperature at 52°C ± 0.5°C. The time of hind paw withdrawal, flinching, licking, or jumping behavior from the plate was recorded. The rats were removed from the hot plate surface once a reaction was observed or after a 20-s cut-off time if no response was observed to prevent tissue damage at 52°C. Each animal was tested twice, separated by a 30-min interval between them (Batista et al., 2019).

#### 2.7.3 Horizontal ladder test

The horizontal ladder walking test was used to evaluate the sensorimotor function following SCI. Before the surgery, the rats were trained to cross a ladder on a regular rung pattern in the same direction. The test was measured at 4 weeks after the induction of SCI. Briefly, each experimental animal was allowed to cross a 1-m-long ladder rod designed with a 1-cm spacing between round metal rods. The run was recorded by a camera. The qualitative evaluation of hind-limb placement was based on a foot scoring system using a 7-category scale (0–6 points). The rating scale (Metz and Whishaw, 2009) (0: total miss, 1: deep slip, 2: slight slip, 3: replacement, 4: correction, 5: partial placement, and 6: correct placement) was used for the quantitative measurement of hind-limb position and foot errors that occurred in placement accuracy.

### 2.8 Histopathology

The rats were killed 4 weeks after SCI. Subjects were deeply anesthetized and transcardially perfused with a 4% solution of paraformaldehyde, and the spinal cord was dissected out of the spinal column. The spinal cords from the experimental rats were fixed in 10% buffered formaldehyde solution and then dehydrated in different grades of ethanol. Tissues were embedded in paraffin wax, and sections of 7 μM thickness on slides were obtained. The tissue sections were stained with toluidine blue and studied by light microscopy. There were three animals per group. The mean values of the five non-serial, paraffin-embedded sections from each animal at the same level of the injured spinal cord (the lesion epicenter) in every 60th slide corresponding to every 300 m along the cord were counted using ImageJ software. All moto-neurons were counted in each section of the embedded spinal cord specimen. The number of α-motoneurons was analyzed by their position in the spinal cord (ventral horn, lamina IX) and size. Only neurons with a diameter >25 μm and a visible nucleus were counted (Chai et al., 2011) (substantially larger than interneurons and glial cells). Two skilled pathologists unaware of the study groups conducted investigations to examine the number of ventral-moto neurons and histological alterations. All the slides were coded to eliminate any bias throughout the experiment, and histological techniques were carried out after combining sections from several groups. Sections were qualitatively assessed using light microscopy for tissue degradation, inflammatory cell infiltration, and hyperemia signs. Luxol Fast Blue staining was used to identify myelinated areas and residual spared tissue in the injured spinal cord segment. The percentage of spared white matter was calculated by dividing the total area of spared white matter by the total area of the spinal cord volume. Finally, an experienced pathologist, blinded to the animal experiment, performed analyses and scored the section for the absence (grade 0) or presence (grade 1, 2, or 3) of tissue degeneration, demyelination, inflammatory cell infiltration, and hyperemia, where the histological score indicates 0=(negative); 1=(mild); 2=(moderate); and 3= (extensive) (Letaif et al., 2015).

### 2.9 Statistical analysis

Data were collected and stored in a database. The statistical analysis was performed on data using GraphPad prism (version 7; GraphPad Software, San Diego, CA). All the data were assessed for homogeneity of variance using the Brown–Forsythe test, where no significant differences among standard deviations were found. Data normality assessment using the Shapiro–Wilcoxon test and further statistical analysis using the Kruskal–Wallis test followed by Dunn’s post hoc test were performed. The BBB locomotor rating scale scores and threshold response were analyzed using two-way analysis of variance (ANOVA) with repeated measures followed by Tukey’s multiple comparisons post hoc test for multiple comparisons between the groups. In case of mRNA and protein level expression, one-way ANOVA followed by Tukey’s post hoc test was used to compare differences between the groups in behavioral results. Data are represented as mean ± SD and were considered statistically significant if *p* < 0.05.

## 3 Results

### 3.1 Expression level of NKCC1 following spinal cord injury

**FIGURE 2:**
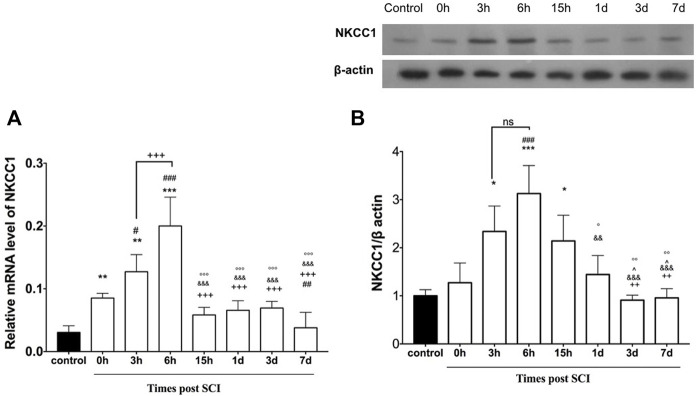
Altered expression of NKCC1 in the lesion center following spinal cord injury. **(A)** Time course-dependent change of mRNA NKCC1. **(B)** Protein expression of NKCC1 at the injured spinal cord (epicenter). *p ≤ 0.05, ** *p* ≤ 0.01, *** *p* ≤ 0.001 vs. control group; # *p* ≤ 0.05, ### ≤ 0.001 vs.0 h group. +++*p* ≤ 0.001 vs. 6 h group. & *p* ≤ 0.001 vs. 3 h group. Data are presented as the mean ± S.D.

To determine the appropriate time to initiate treatment after SCI, here we assessed the mRNA and protein expression level of NKCC1 at the injured spinal cord following different time points post SCI. To assess the time-dependent differences between the mRNA and protein expression level of NKCC1 at the lesion center, the rats were subjected to contusion spinal cord injury. Then, the lesioned area was isolated at distinct timepoints to determine both mRNA and protein expression level using real-time PCR and Western blot, respectively. The pattern of changes at the NKCC1 level was determined (Figure 2A). One-way ANOVA demonstrated significant differences in the mRNA level [F (7, 51) = 39.45, *p* < 0.0001] following SCI. Upregulation of the mRNA level was detected immediately after injury (0 h) compared to the intact group. Importantly, the mRNA level was significantly increased at 3 h and peaked around 6 h after SCI, (*p* < 0.001). An immunoblotting study was conducted to evaluate the NKCC1 protein levels of the spinal cord in the time course after SCI. Verification of the assumption of normality was assessed by the Shapiro–Wilk test (*p* < 0.05). One-way ANOVA showed significant difference between the groups [F (7, 16) = 12.01, *p* < 0.0001]. NKCC1 protein expression altered from the initial time of injury to 24 h post SCI. The protein levels of NKCC1 rapidly elevated after injury compared to those of the control group, peaked at 6 h, and was maintained at high levels on 15 h but reduced until 7 days after SCI but increased at 28 days. Our data showed that the NKCC1 protein levels upregulated up to threefold on 6 h and approximately twofold on 3 and 15 h after injury (Figure 2B). According to these results, we have chosen the 3-h timepoint for NKCC1 blockade after SCI for the following experiment.

### 3.2 Early inhibition of NKCC1 improves spontaneous recovery of locomotor function

**FIGURE 3:**
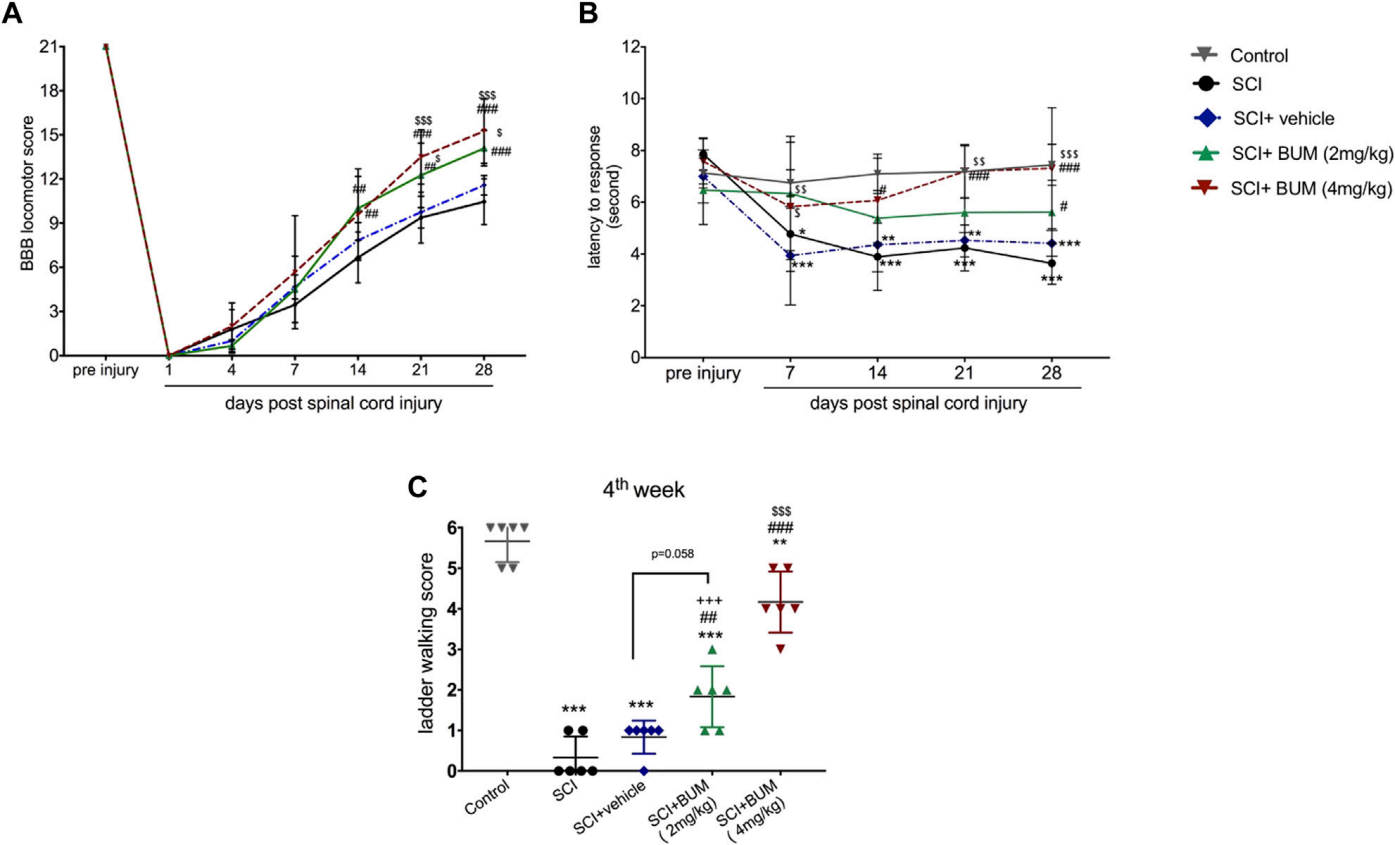
Beneficial effect of early and repeated intraperitoneal administration of bumetanide on functional recovery after SCI. **(A)** Improvement in the BBB scale after SCI in the bumetanide treatment group. The Bum 4 mg/kg rats demonstrated superior BBB score with significant recovery than the SCI and vehicle group. **(B)**. Analgesic effect of early bumetanide treatment on thermal hyperalgesia in rats with contusion spinal cord injury. Bumetanide reversed thermal hyperalgesia induced by SCI during 28 days. **(C)** Foot fault scoring of rats on the horizontal ladder walking test analyzed using qualitative ratings for limb placement and accuracy at 4 weeks after SCI. Both bumetanide treatment groups showed significant differences from the SCI group. Bum 4 mg/kg showed the highest score at 4 weeks. * *p* ≤ 0.05, ** *p* ≤ 0.1, *** *p* ≤ 0. 001 vs. control group; ## *p* ≤ 0.01, ### *p* ≤ 0 001 vs. the SCI group; $ *p* ≤ 0.05, $$ *p* ≤ 0.01, $$$ *p* ≤ 0.001 vs. the vehicle group. Data are presented as the mean ± S.D.

We first studied the impact of early bumetanide treatment on locomotor function. The rats were given bumetanide at concentrations of 2 and 4 mg/kg or vehicle once daily by intraperitoneal injection for 28 consecutive days starting 3 h after contusion spinal cord injury. BBB scoring is used to evaluate motor performance of the rats. Two-way ANOVA with repeated-measures for the BBB score demonstrated a significant effect of treatment [F (3.15) = 5.67, *p* = 0.008), time (F (6.30) = 730.7, *p* < 0.0001] and treatment × time interaction [F (18,90) = 4.24; *p* < 0.0001]. Prior to spinal cord injury, all the animals showed normal hind-limb function. At day 1 post spinal cord injury, all animals presented complete paralysis of hind-limb function. All the animals show spontaneous motor recovery up to 4 weeks. However, the rats in both bumetanide treatment groups showed significantly better spontaneous motor recovery than those in the SCI group starting from day 14 (*p* < 0.01). Furthermore, the Bum 2 mg/kg group and the Bum 4 mg/kg group had a higher score than the vehicle group from day 21 (*p* < 0.05, *p* < 0.001, respectively). No significant differences were observed between the vehicle groups and the SCI group. (Figure 3A). These data suggest that 3 h post injury is a proper time for improvement of motor function.

### 3.3 Early bumetanide treatment ameliorates hyperalgesia in contusion spinal cord injury rats

Next, we investigated whether repetitive daily treatment with bumetanide (2 or 4 mg/kg) would alter the nociceptive threshold upon thermal stimulation compared with no bumetanide treatment groups. We found that the withdrawal response for thermal stimuli was significantly changed for rats in both the SCI and vehicle-treated groups (Figure 3B). Two-way ANOVA with repeated-measures demonstrated a significant effect of treatment [F (4,24) = 14.97, *p* < 0.0001), time (F (4, 24) = 21.1, *p* < 0.0001) and treatment × time interaction [F (16, 96) = 2.801; *p* = 0 .001] on paw withdrawal threshold. In both SCI and vehicle groups, withdrawal latency decreased after 7 days, and this phenomenon continued till 28 days after injury, which indicates hyperalgesia. However, this phenomenon was ameliorated by bumetanide 2 mg/kg but did not show significant improvement. On the other hand, 4 mg/kg bumetanide significantly improved hyperalgesia. This result indicates that 4 mg/kg bumetanide after SCI improved pain sensation.

### 3.4 Early bumetanide treatment could restore hind-limb sensorimotor function

An assessment of the sensorimotor coordination was performed at 4 weeks following SCI using the ladder-walking test. After SCI, sensorimotor function was disrupted. The animals in the SCI and vehicle groups were not able to cross the ladder and were scored with 0–1 point, which was significantly different from the Bum 4 mg/kg group (*p* < 0.001). The rats in the Bum 2 mg/kg group were occasionally able to cross the ladder, which resulted in the final score of 1.833 ± 0.75. The rats in the Bum 4 mg/kg group were able to cross the ladder with some missteps and achieved a score of 4.167 ± 0.75. No significant difference between the Bum 2 mg/kg and vehicle group was observed (Figure 3C).

### 3.5 Bumetanide downregulates NKCC1 expression in spinal cord injury rats

**FIGURE 4:**
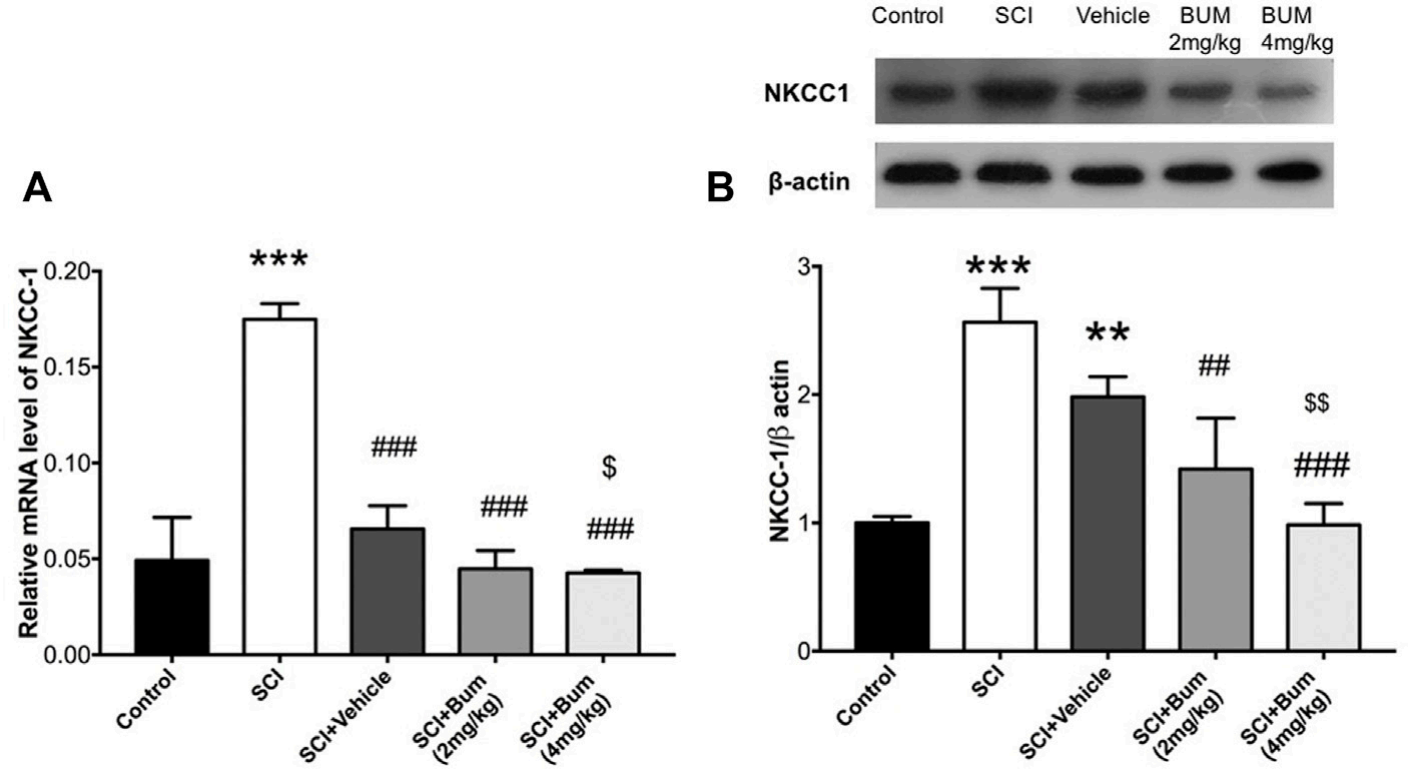
Bumetanide suppresses NKCC1 expression. **(A)** NKCC1 gene expression levels of the injured spinal cord at 4 weeks after SCI. Related gene expression levels were upregulated at the SCI group, and Bum 4 mg/kg treatment decreased the NKCC1 expression compared to the vehicle. **(B)** Representative Western blots of and quantitative data for NKCC1 and b-actin expression in each SCI group. NKCC1 protein expression level was decreased by bumetanide treatment. ** *p* ≤ 0.01, *** *p* ≤ 0.001 vs. control group; ## *p* ≤ 0.01, ###*p* ≤ 0.001 vs. the treated SCI group. $ *p* ≤ 0.05, $$ *p* ≤ 0.01 vs. vehicle group. Data are presented as the mean ± S.D. Loading controls were reused across Figures 4 and 5 of this article.

The rats received bumetanide (2 mg/kg and 4 mg/kg) as an NKCC1 inhibitor, 3 h after SCI. The mRNA and protein expression levels of NKCC1 were evaluated in the injured spinal cord segment following long-term bumetanide administration for 28 days. One-way ANOVA of the results revealed significant differences between groups [F (4,21) = 83.08, *p* < 0.0001]. We found that the mRNA expression level of NKCC1 at the lesion site of the spinal cord in un-treated SCI groups markedly increased (Figure 4A). In contrast, the mRNA level of the SCI group treated with bumetanide (4 mg/kg per day, starting 3 h after contusion for 28 days) was significantly decreased as compared with those of the vehicle and un-treated SCI groups.

Verification of the assumption of normality was assessed by the Shapiro–Wilk test (*p* < 0.05). Afterward, one-way ANOVA of the results revealed significant differences between groups [F (4,10) = 24.53, *p* < 0.0001]. Western blot analysis indicated that SCI was accompanied by upregulation of NKCC1 protein levels in comparison to control, and also our data showed that blocking of NKCC1 resulted in a significant decrease of its protein levels compared to the SCI-untreated group (Figure 4B). In other words, bumetanide leads to inhibition of SCI-induced NKCC1 overexpression. Also, it was observed that vehicle administration reduced the NKCC1 protein levels, but there were no significant differences compared to the untreated group.

### 3.6 Effect of bumetanide on GAP-43 expression in spinal cord injury rats

**FIGURE 5:**
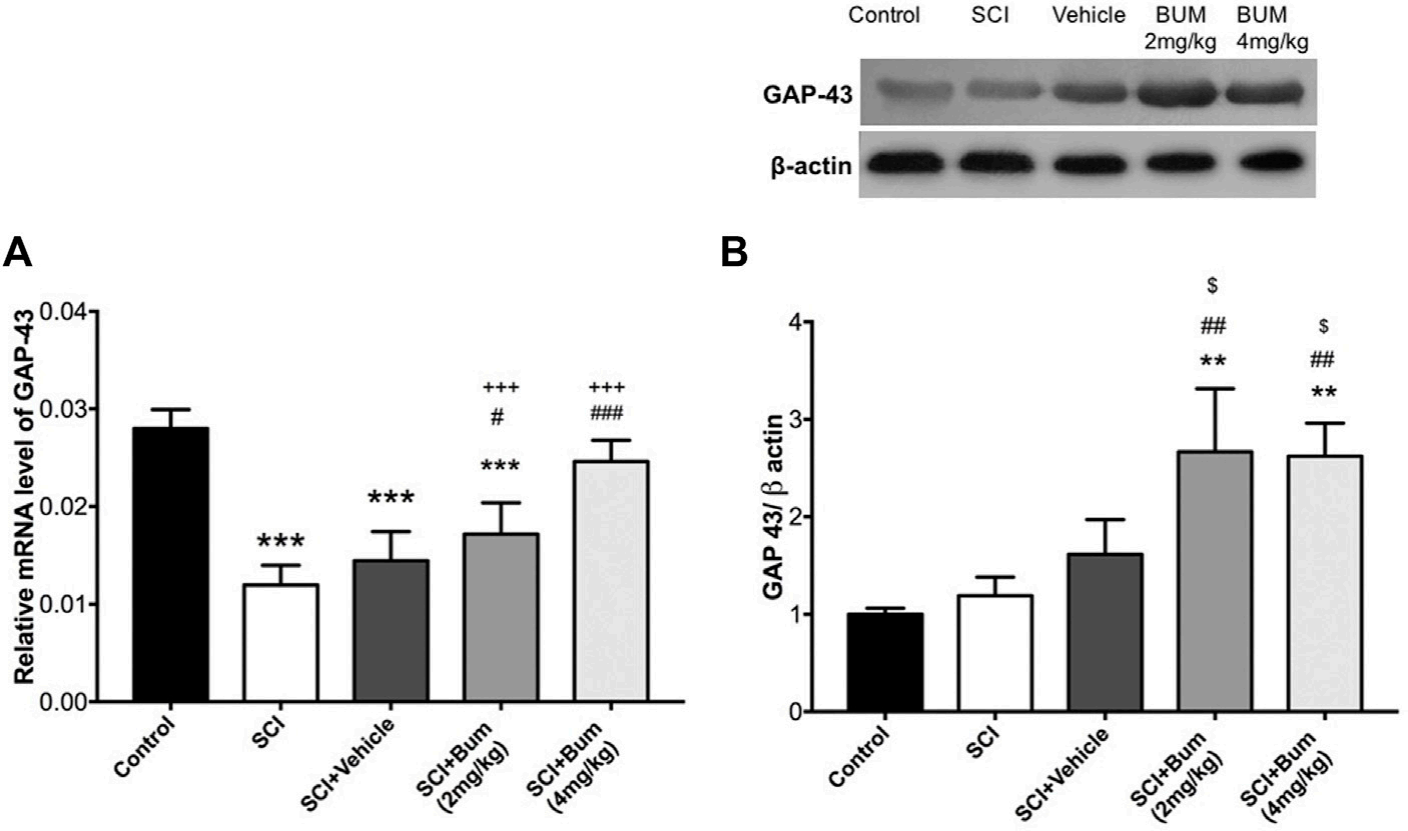
**(A)** GAP-43 gene expression levels of the injured spinal cord at 4 weeks after SCI. Bumetanide enhanced the GAP-43 neurogenesis-related gene expression levels in a dose-dependent manner. **(B)** Representative Western blots and quantitative data GAP-43 and b-actin expression in the injured segment. Bumetanide increased the GAP-43 marker protein expression levels. *** *p* ≤ 0.001 vs. the control group; # *p* ≤ 0.05, ## *p* ≤ 0.01, ### *p* ≤ 0.001 vs the SCI group; +++*p* ≤ 0.001 vs. Bum 4 mg/kg group; $ *p* ≤ 0.05 vs. the vehicle group. Data are presented as the mean ± S.D. Loading controls were reused across Figures 4 and 5 of this article.

To evaluate whether the blocking of NKCC1 may induce repair, the mRNA and protein expression levels of GAP-43 were measured in the spinal cord samples following 28-day administration of bumetanide. One-way ANOVA indicated significant differences in the protein level [F (4,22) = 36.22, *p* < 0.0001] on day 28 after SCI. Significant reductions in the mRNA level of GAP-43 were observed in the SCI, vehicle, and BUM 2 mg/kg group compared to the control rats. Treatment with BUM 4 mg/kg after 28 days showed no difference in the mRNA level of GAP-43 (Figure 5A). Verification of the assumption of normality was assessed by the Shapiro–Wilk test (*p* < 0.05). Afterward, one-way ANOVA indicated significant differences in the protein level [F (4,10) = 13.24, *p* = 0.0005] on day 28 after SCI. Our data indicated there was no significant difference between the protein expression of GAP-43 on the control, un-treated SCI, and vehicle groups. However, bumetanide administration (2 mg/kg and 4 mg/kg) markedly upregulated GAP-43 protein levels in the injured spinal cord (Figure 5B).

### 3.7 Effects of bumetanide on histological changes

**FIGURE 6:**
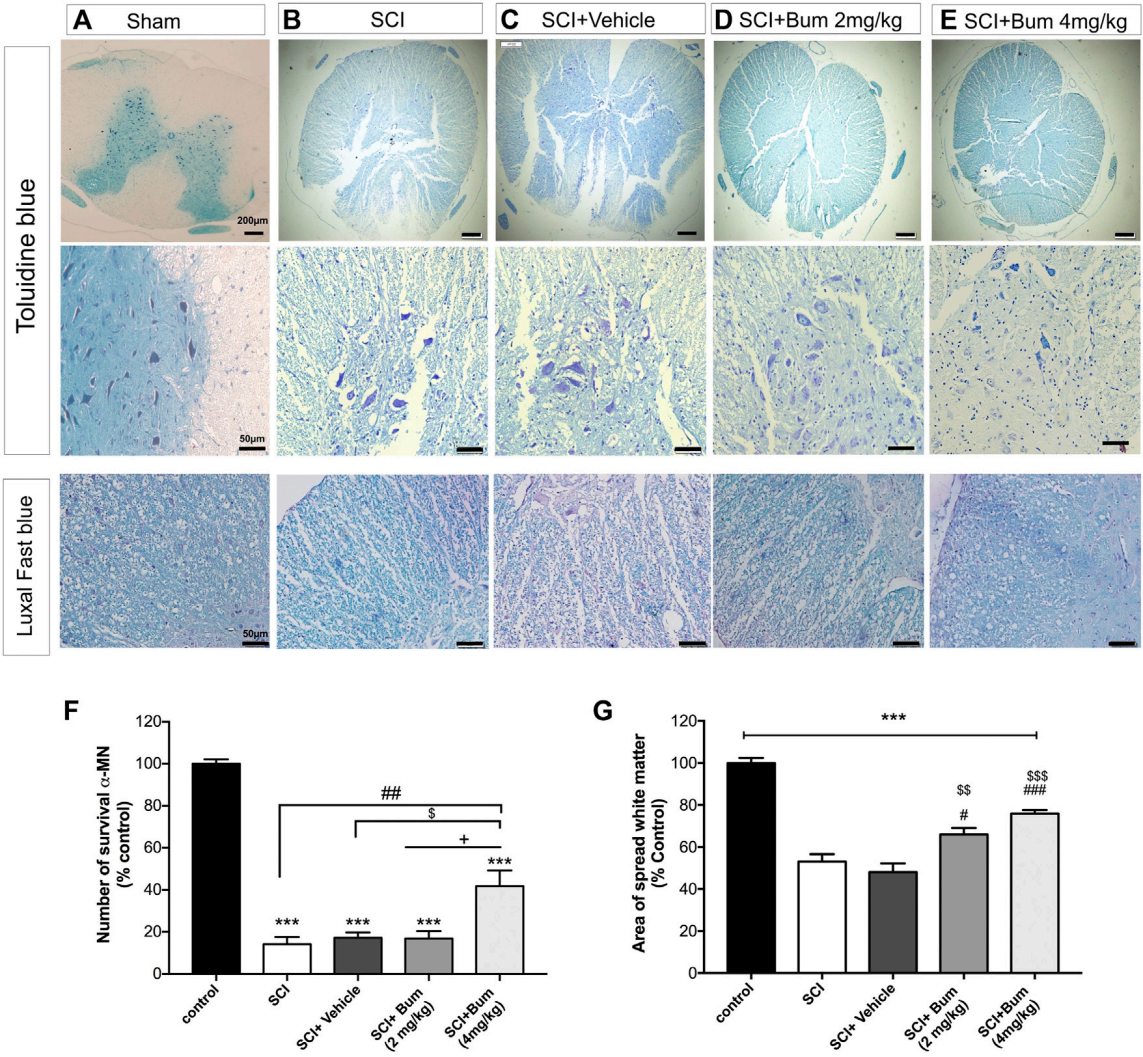
Toluidine blue staining for rats undergoing spinal cord injury (SCI) evaluated at 28 days post SCI **(A–D)**. Histopathologic features were investigated at high magnification, region of extensive degeneration (red box, panel b–c) with demyelination (red arrows, panels b–c), and infiltration (black arrow panel b). **(F)** Quantitative analysis for the number of motor neurons (% of sham group) among the groups was determined at 4 weeks after SCI. **(G)** Graph bar shows quantitative evaluation of spared white matter in LFB-stained sections. The bumetanide 4 mg/kg group showed more white matter than the SCI group. The data are shown as the mean ± SD. *** *p* ≤ 0.001 vs. control, # *p* ≤ 0.05, ## *p* ≤ 0.01, vs. the treated SCI group; $ *p* ≤ 0.05, $$ *p* ≤ 0.01, $$$ *p* ≤ 0.001 vs. the vehicle group; +*p* ≤ 0.05 vs. Bum 2 mg/kg group; scale bar = 50 μm.

We performed further investigation to see whether changes in the GAP-43 expression are associated with histological changes. Histopathological changes in spinal cord tissue are shown in Table 1. The spinal cord of the SCI (un-treated) group showed major structural abnormalities consisting of degeneration of spinal cord tissue, demyelination, infiltration of inflammatory cells, and hyperemia (Figure 6B). In the SCI+ bumetanide 4 mg/kg group, hyperemia and infiltration of inflammatory cells to the injury site significantly diminished compared to the bumetanide 2 mg/kg group (Figures 6D,E). In addition, the bumetanide 4 mg/kg reduced degenerative changes of spinal cord tissue. In the vehicle group (ethanol), degeneration on the spinal cord tissue similar to the SCI group was observed (Figure 6C). In addition, motor neurons from the SCI (Figure 6B) and vehicle (Figure 6C) rats show pathological shrinkage which was reduced by Bum 4 mg/kg treatment (Figure 6E). To evaluate the effect of SCI on the survival of motor neurons, the total number of pyramidal neurons of the ventral horn was counted. One-way ANOVA analysis indicated a significant difference between the number of motor neurons [F (4,15) = 47.23; *p* < 0.0001] in the injured spinal cord. A significantly increased number of motor neurons was found in the Bum 4 mg/kg group compared to all the SCI, vehicle, and Bum 2 mg/kg groups 28 days post SCI. (Figure 6F). Quantification of the spared white matter showed significantly better preservation in both the bumetanide treatment groups and had a positive effect on tissue preservation than the SCI and vehicle groups. The control group showed a normal pattern of white matter distribution. Statistical analysis indicated the area of preserved tissue after SCI at the lesion site which was less (53%) than in the control group. The low percentage of preserved white matter was also detected in the vehicle group (48%), while both the treatment groups exhibited less white matter area (WMA) loss at the lesion site in the same segments. In the 4th week of survival, the preserved white matter was better, and it reached 65% and 75%. (Figure 6G). Bumetanide groups revealed better improvement in histological and functional features which could be explained by the reduced moto-neuronal loss and the preserved white matter sparing in the injured spinal cord. We assume bumetanide therapy was associated with significant white matter tissue survival in the injured segment.

**TABLE 1:** Comparison of histological changes of late SCI, such a hyperemia, degeneration, cellular infiltration, and demyelination between SCI and treatment groups at 4 weeks post SCI. Histological score: 0= (negative); 1= (mild); 2= (moderate); 3= (severe).

Group	SCI	SCI+ Vehicle	SCI+ Bum 2 mg/kg	SCI+ Bum 4 mg/kg
Hyperemia	1	1	1.5	0
Degeneration	3	2	2	0.5
Infiltration	3	2	2	1
Demyelination	2	2	2	1

## 4 Discussion

The upregulation of NKCC1 and the subsequent shift of GABA_A_ to excitatory functions is a fundamental transformation shown in some nervous diseases, such as autism, temporal lobe epilepsy (Ben-Ari et al., 2012; Gharaylou et al., 2019a), schizophrenia (Kehrer et al., 2008), neuropathic pain (Côté, 2020), and animal models of the Down syndrome (Potier et al., 2014). Bumetanide is an NKCC1 inhibitor which showed promising effects in improving the conditions associated with these circumstances (Ben-Ari, 2017; Gharaylou et al., 2019c; Zarepour et al., 2020). The present study analyzes bumetanide’s effect on SCI rat recovery using the BBB score, horizontal ladder walking, and the hot plate test for assessment of motor neuron function, sensorimotor function, and sensory evaluation, respectively. Moreover, histological and molecular assessments were used to validate these measurements. Therapeutic effects of bumetanide (4 mg/day) became visible 28 days after SCI when compared to those of baseline. There are three major types of spinal cord injury: compression, transaction, and contusion (Ahmed et al., 2019). Contusion was the most common pattern of injury (41%), followed by transection (32.5%) and compression (19.4%) (Sharif-Alhoseini et al., 2017a). On the basis of the literature, thoracic SCI models are apparently reliable and easy to reproduce (Rahimi-Movaghar, 2009). Most studies used a blunt trauma injury pattern such as contusion, compression, distraction, dislocation, or existing traumatic SCI (Sharif-Alhoseini et al., 2017b). Histological data revealed the typical characteristics of contusion (Nishi et al., 2007). The lesion site was composed of hemorrhage, edema, and infiltration by inflammatory cells and cystic microcavitations, as seen in human SCI (Baussart et al., 2006). Our study showed that the aforementioned pattern was found in our rat SCI model.

The widespread expression of GABAergic neurons in the spinal cord and their role in the early and late phases of SCI deserve full attention. The gradient of intracellular and extracellular chloride ions has changes in mature and immature GABAergic neurons. Spinal cord lesions disrupted the state of balanced networks like immature neurons because of the downregulation of KCC2 or upregulation of NKCC1 (Mazzone et al., 2021). Consistent with preceding reports, our study further presented evidence of the change of NKCC1 expression in the lesion after spinal cord injury. NKCC1 was overexpressed 3 h after trauma, peaking at hour 6 and persisting at an elevated level for 7 days. Consequently, in order to inhibit NKCC1, 3 hours after SCI was selected as an appropriate timepoint for bumetanide treatment. We tried to simulate the time in which paramedics can reach an injured patient, and it is exactly the time when the NKCC1 expression is upregulated. The rats received bumetanide 3 h after SCI for 28 days. Disturbance of the BBB score after induction of spinal cord injury has been well-shown by previous studies. Proper time of injection of bumetanide improves the BBB scores, indicating that this drug prevents chloride disturbance and GABA dysfunction in motor neurons injured in SCI. Consistent with our results, a study has shown that the locomotor network was improved by applying anodal trans-spinal direct current stimulation plus bumetanide after spinal cord contusion (Mazzone et al., 2021). Another study reported that bumetanide improved behavioral outcomes after cerebral ischemia (Mu et al., 2017). Some studies injected bumetanide before induction of injury, which is not in accordance of this natural disease (Mazarati et al., 2009; Yan et al., 2018). We try to design our study based on what happens clinically. The GABA-mediated depolarization through upregulation of NKCC1 contributes to secondary post damage, edema, neuron death, and destruction of spinal cord tissues (Mazzone et al., 2021). The failure of spontaneous neurite regeneration and neurological connectivity restoration leads to persistent functional deficits following SCI. The GABA-mediated depolarization inhibits axonal regeneration by stimulating the Rho/Rock signaling pathway and the pan-neurotrophin receptor p75NTR synthesis, increasing cell death (Shulga et al., 2012). Although changes in the BBB scores are important, if these changes were not associated with the cellular and histological improvement, they may not be of long-standing benefit. Our histological analysis revealed that 4 mg/kg bumetanide was the best dosage to overcome histological changes after SCI and induced reconstruction in injured spinal cord. Bumetanide also prevented pathological upregulation of p^75NTR^ and neuronal death in the injured areas (Shulga et al., 2012). Peripheral nerve regeneration and healing was obtained by the synergistic effect of dexamethasone and bumetanide on edema and aquaporin 1 receptor (Longur et al., 2021). GAP-43 is an intrinsic determinant of neuronal development and plasticity. Histological assessment of our study showed a greater diameter of neurons and reduced white matter loss after bumetanide treatment, which may be due to preservation of the myelin sheath (Wang et al., 2018). Western blotting analysis in our study confirmed that bumetanide promotes the expression of GAP-43 levels. Physiological dysfunction is one of the major features in spinal cord injury. Researchers in this field try to ameliorate these dysfunctions, such as hyperalgesia and sensory motor dysfunction. Previous research studies showed NKCC1 upregulation following peripheral nerve injury leads to allodynia and hyperalgesia. In addition, KCC2 downregulation due to overexpression of Brain-Derived Neurotrophic Factor has also caused complicated pain states (Kahle et al., 2008). Restoring these patterns and pharmacological manipulations of these co-transporters has pronounced implications for controlling neuropathic pain and providing efficient analgesic effects (Gwak and Hulsebosch, 2011; Mòdol et al., 2014). Injection of 4 mg/kg bumetanide controlled the aforementioned dysfunctions and restored physiological properties. The bumetanide 4 mg/kg group achieved faster recovery of the hind-limb function at 4 weeks in comparison with the non-treated and vehicle animals. It seems that the preservation of axons might be essential for a favorable neurological outcome.

This study is a preliminary study to examine the effect of bumetanide treatment in the contusion SCI rat. Previous studies revealed that different recovery was observed between male and female rats after the spinal cord injury (Datto et al., 2015). One limitation of this study is only male rats were used. To provide more scientific evaluation which allows potential translation to humans, further research on female rats is crucial. Pre-clinical studies on both sexes as a biological variable will help examine the long-term effect of bumetanide treatment for clinical application.

## 5 Conclusion

This study provides novel evidence for the benefits of bumetanide after SCI. Further investigation by using a primate model will confirm whether bumetanide as an FDA-approved drug can control all adverse effects of spinal cord injury. Thereafter, it may also be part of the protocol for acute SCI care.

## Data Availability

The original contributions presented in the study are included in the article/Supplementary Materials; further inquiries can be directed to the corresponding author.
